# *Bombyx mori* RPL12 Participates in UV-Induced DNA Damage Repair and Interacts with BmNPV Bm65 Protein Only After Ultraviolet Radiation

**DOI:** 10.3390/insects16020187

**Published:** 2025-02-09

**Authors:** Qi Tang, Ceru Chen, Jiaying Huang, Guohui Li, Feifei Zhu, Qian Yu, Lindan Sun, Huiqing Chen, Liang Chen, Shangshang Ma, Xiaoyong Liu, Keping Chen

**Affiliations:** School of Life Sciences, Jiangsu University, 301# Xuefu Road, Zhenjiang 212013, Chinaqianyu@ujs.edu.cn (Q.Y.); sunlindan@ujs.edu.cn (L.S.);

**Keywords:** *Bombyx mori*, BmNPV, ultraviolet radiation, DNA damage repair, protein-protein interaction

## Abstract

Solar ultraviolet radiation (UV) may cause DNA damage. We first report in this study that the large subunit ribosome protein RPL12, from *Bomby mori* (BmRPL12), participated in UV-induced DNA damage repair. BmRPL12 enhanced the resistance of *Escherichia coli* (*E. coli*) to UV radiation and facilitated faster repair of UV-induced DNA damage in silkworm cells. BmRPL12 mainly existed in the cytoplasm in the dimer forms, and the N-terminal nuclear export signal was crucial for the localization of BmRPL12. After UV radiation, BmRPL12 was unable to localize at the UV-induced DNA damage sites to participate in damage repair directly and might indirectly regulate UV-induced DNA damage repair. Our previous research found that BmNPV Bm65 was an important UV damage-induced endonuclease. Here, it was further found that in BmNPV-infected silkworm cells, BmRPL12 in monomeric forms interacted with the virus Bm65 protein only after UV radiation, and BmRPL12 specifically localized at the UV-induced DNA damage sites only in the presence of Bm65. We speculate that after viral infection in cells subjected to UV-induced DNA damage, viral protein Bm65 interacts with BmRPL12 and localizes BmRPL12 to sites of UV-damaged DNA.

## Simple Summary

Because of environmental pollution, solar ultraviolet radiation is increasing day by day and typically causes DNA damage. Here, we found that a *Bombyx mori* ribosomal protein RPL12 is indirectly involved in UV-induced DNA damage repair. BmNPV Bm65 protein selectively interacts with host protein BmRPL12, alters the localization of BmRPL12, and collaborates with this host protein to directly participates in UV-induced DNA damage repair. These results provide a reference for further research on the DNA damage repair mechanism in host insects and viruses.

## 1. Introduction

Because of environmental pollution, solar ultraviolet radiation is increasing day by day. UV radiation typically causes DNA damage, including 75% cyclobutane pyrimidine dimers (CPDs) and 25% pyrimidine 6-4 pyrimidone photoproducts. Base excision repair and nucleotide excision repair play an important role in UV-induced DNA damage repair [1]. In addition, photoreactivation uses photolyases to repair UV-damaged DNA [2].

Ribosomal proteins are composed of proteins located in the large subunit of the ribosome (RPL) and proteins located in the small subunit of the ribosome (RPS), which are involved in protein synthesis. More and more reports have shown that ribosomal proteins have multiple functions, including protein biosynthesis, DNA repair, cell differentiation, and cell development regulation [3,4,5,6]. RPL12 is a highly conserved protein from yeast to humans [7]. Plant ribosomal protein RPL12 was reported to play a role in nonhost disease resistance against bacterial pathogens [8]. RPL12 was also implicated in the dynamic coordination of ribosome biogenesis [9].

*Bombyx mori* is an important Lepidopteran insect in agriculture. Baculoviruses are enveloped double-stranded DNA viruses that primarily infect insects from the orders Lepidoptera, Hymenoptera, and Diptera [10]. *Bombyx mori* nucleopolyhedrovirus (BmNPV) belongs to the family *Baculoviridae* and specifically infects *Bombyx mori*. BmNPV infection disturbed the insect hormone balance [11]. The infection of BmNPV causes about 20% loss of cocoon in the silk industry annually [12]. In this study, *Bombyx mori* RPL12 (BmRPL12) was first found to contribute to UV-induced DNA damage repair. When there was no viral infection, BmRPL12 mainly existed in the cytoplasm in the form of dimers and indirectly regulated UV-induced DNA damage repair. In our preliminary research, we discovered that BmNPV Bm65 was an important UV endonuclease and beneficial for UV-induced DNA damage repair of the host [13,14]. At present, the UV damage repair mechanism of most baculoviruses is still unclear. Based on the important role of Bm65 in UV damage repair, the interacting proteins of Bm65 were further identified through Co-Immunoprecipitation (Co-IP) assays combined with Mass spectrometry analysis, including host protein BmRPL12. Interestingly, we only detected interaction between BmRPL12 and Bm65 in the presence of UV radiation (unpublished data). Viral infection often affects the expression and function of host proteins. To explore how host proteins and viral proteins selectively interact under different conditions, in this study, BmRPL12 was further validated to interact with Bm65 in monomeric form at the UV-damaged DNA sites only after UV radiation. It was speculated that in BmNPV-infected cells, BmRPL12 might directly participate in UV damage repair through Bm65-mediated pathways. These results provided a reference for further research on the DNA damage repair mechanism in host insects and baculoviruses.

## 2. Materials and Methods

### 2.1. Plasmids and Cells

The plasmids pHTB-Pie-1-gfp, pHTB-Pie-1-Bm65-gfp, and pHTB-Pie-1-mCherry were constructed in our laboratory [14,15,16]. Bm65-deleted BmNPV (vBm^Bm65KO^) and repair-type viruses (vBm^Bm65-flag^) were constructed previously [17]. BmN cells were cultured at 27 °C in TC-100 insect medium supplemented with 10% fetal bovine serum (Gibco/Thermo Fisher Scientific, Waltham, MA, USA). All primers used in the study were listed in Table 1.

### 2.2. Effects of BmRPL12 on the UV Sensitivity of E. coli

BmRPL12 was amplified by PCR and ligated with pET30a to construct the recombinant plasmid pET30a-BmRPL12. The *E. coli* cells (BL21) were transformed with the recombinant plasmid pET30a-BmRPL12 or control plasmid pET30a. Then, the cells were, respectively, spread onto LB agar plates and exposed to different doses (0.15 J/m^2^, 0.3 J/m^2^) of UVC light generated by a hand-held wand (Thermo, Waltham, MA, USA). The number of colonies was counted after cultivation in the dark, and the percent survival was calculated as the ratio of UV-treated to untreated BL21 cells. Statistical analysis was performed using single-factor analysis of variance.

### 2.3. Localization Analysis of BmRPL12 in BmN Cells

Primers BmRPL12-mCherry-F and BmRPL12-mCherry-R were used to construct pHTB-Pie-1-BmRPL12-mCherry (1–179 aa). Primers BmRPL12-mCherry-F and BmRPL12-T1-mCherry-R were used to construct pHTB-Pie-1-BmRPL12-T1-mCherry (1–110 aa). Primers BmRPL12-T2-mCherry-F and BmRPL12-mCherry-R were used to construct pHTB-Pie-1-BmRPL12-T2-mCherry (97–179 aa). The related truncated method referred to previous research reports [18]. The cells were transfected with the above recombinant plasmids and cultured for 48 h at 27 °C. After fixed with 4% paraformaldehyde and permeabilized in 0.1% Triton X-100, the transfected BmN cells were treated with DAPI (Sigma, Dorset, UK) and photographed using a fluorescence microscope (Leica, Wetzlar, Germany).

### 2.4. Knockdown of BmRPL12 in Silkworm Cells

BmN cells were separately transfected with 20 μM BmRPL12 siRNA or control siRNA (GenePharma, Suzhou, China) using Cellfectin II reagent (Invitrogen, Carlsbad, CA, USA) to knock down the expression of BmRPL12. The knockdown efficiency was verified by Western blotting at 48 h after transfection and ImageJ software 1.54f was further used to quantify the western blot bands. Anti-RPL12 rabbit polyclonal antibody (1:500, TransGen Biotech, Beijing, China) was used to detect the protein expression level of BmRPL12. Anti-α-Tubulin rabbit monoclonal antibody was used for loading control (1:1000, Cell Signaling Technology, Danvers, MA, USA). The goat anti-rabbit IgG H&L (HRP) (1:1000, TransGen Biotech, Beijing, China) was used as the secondary antibody.

### 2.5. Effect of BmRPL12 on UV-Induced DNA Damage Repair

BmRPL12 siRNA was transfected into BmN cells to knock down the expression of BmRPL12. After 48 h of cultivation, control cells (transfected with control siRNA) and BmRPL12-knockdown cells were exposed to 60 J/m^2^ UVC. The total DNA of these cells was, respectively, extracted at different time points. The CPD ELISA Kit (Abcam, Cambridge, UK) was used to analyze the UV-induced DNA damage in the extracted DNA. Statistical analysis was performed using single-factor analysis of variance.

### 2.6. Co-Immunoprecipitation (Co-IP) and Western Blotting Analysis

The interaction between BmRPL12 and Bm65 was analyzed by Co-IP assays using anti-RPL12 polyclonal antibodies (TransGen Biotech, Beijing, China) and anti-Flag (TransGen Biotech, Beijing, China) monoclonal antibodies. BmN cells infected with vBm^Bm65-flag^ (MOI = 10) or 60 J/m^2^ UVC-treated BmN cells infected with vBm^Bm65-flag^ (MOI = 10) were, respectively, lysed using RIPA lysis buffer with 10% Phenylmethanesulfonyl fluoride (PMSF) (Beyotime, Shanghai, China) and incubated with the mouse anti-Flag monoclonal antibody (1:500) or rabbit anti-RPL12 polyclonal antibodies (1:200) overnight at 4 °C. Then, the immune complexes were incubated with Protein A/G Sepharose beads (Abcam, Cambridge, MA, USA), eluted with 5× sodium dodecyl sulfate (SDS) loading buffer, and further analyzed by Western blotting. The goat anti-mouse IgG/HRP (1:1000, TransGen Biotech, Beijing, China) and goat anti-rabbit IgG/HRP (1:1000, TransGen Biotech, Beijing, China) were used as secondary antibodies.

### 2.7. Colocalization Analysis of BmRPL12 and Bm65 with UV-Induced DNA Damage (CPD)

The plasmids pHTB-Pie-1-BmRPL12-mCherry and pHTB-Pie-1-Bm65-gfp were transfected into the BmN cells using the Cellfectin II reagent (Invitrogen, Carlsbad, CA, USA). The transfected cells were covered by a UV-opaque polycarbonate filter membrane with 5-μm pores (Millipore, Billerica, MA, USA) and irradiated with 200 J/m^2^ UVC to form local DNA damage. The treated cells were subsequently infected with Bm65-deleted viruses (vBm^Bm65KO^) (MOI = 5). After being fixed with 4% paraformaldehyde and permeabilized with 0.1% Triton X-100, the cells were treated with anti-CPD (CPD, the most prevalent form of UV-induced DNA damage) mouse monoclonal antibody (1:200, Cosmo bio, Japan) and goat anti-mouse IgG H&L Alexa Fluor^®^405 (1:300, Abcam, Cambridge, MA, USA) and photographed using a fluorescence microscope (Leica, Wetzlar, Germany).

## 3. Results

### 3.1. Sequence Analysis of BmRPL12

**Figure 1:**
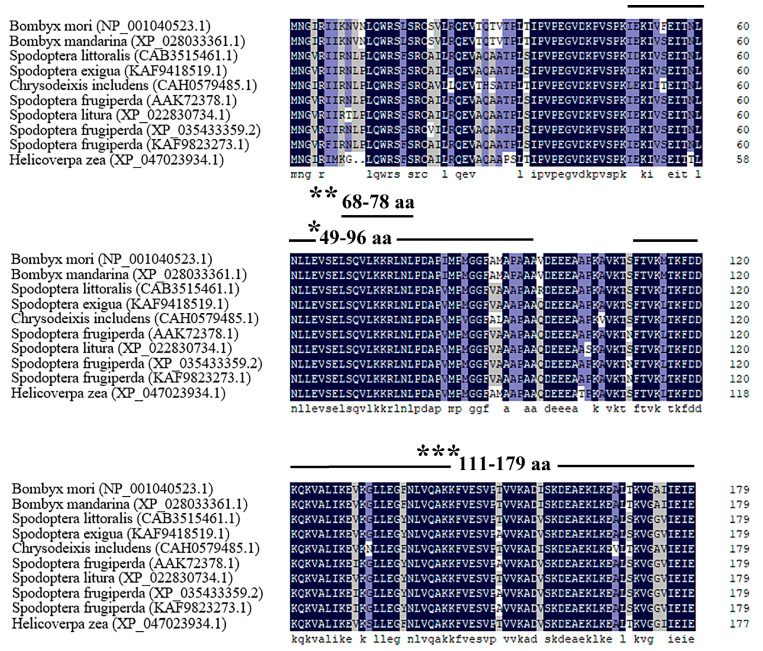
Amino acid alignment of BmRPL12 with its homologous proteins in Lepidoptera. Identical amino acids were denoted by black shading, and similar amino acids were denoted by blue shading (75% similar) or gray shading (50% similar). The amino acid sequence information was from the NCBI database, and the accession numbers were as follows: *Bombyx mori* (NP_001040523.1), *Bombyx mandarina* (XP_028033361.1), *Spodoptera littoralis* (CAB3515461.1), *Spodoptera exigua* (KAF9418519.1), *Chrysodeixis includens* (CAH0579485.1), *Spodoptera frugiperda* (AAK72378.1), *Spodoptera litura* (XP_022830734.1), *Spodoptera frugiperda* (XP_035433359.2), *Spodoptera frugiperda* (KAF9823273.1), and *Helicoverpa zea* (XP_047023934.1). * shows an N-terminal dimerization domain; ** shows an N-terminal nuclear export signal; and *** shows a C-terminal domain, which was predicted to be necessary for the combination of translation factors. Multiple sequence alignment was performed using ClustalW and DNAMAN 9.0.1.116.

The length of BmRPL12 is 537 bp, which codes for a 179 amino acid protein. Multiple sequence alignments of BmRPL12 protein in different species of Lepidoptera showed that BmRPL12 was highly conserved, containing an N-terminal dimerization domain, an N-terminal nuclear export signal, and a C-terminal domain (Figure 1).

### 3.2. BmRPL12 Improved the UV Survival Rate of E. coli

**Figure 2:**
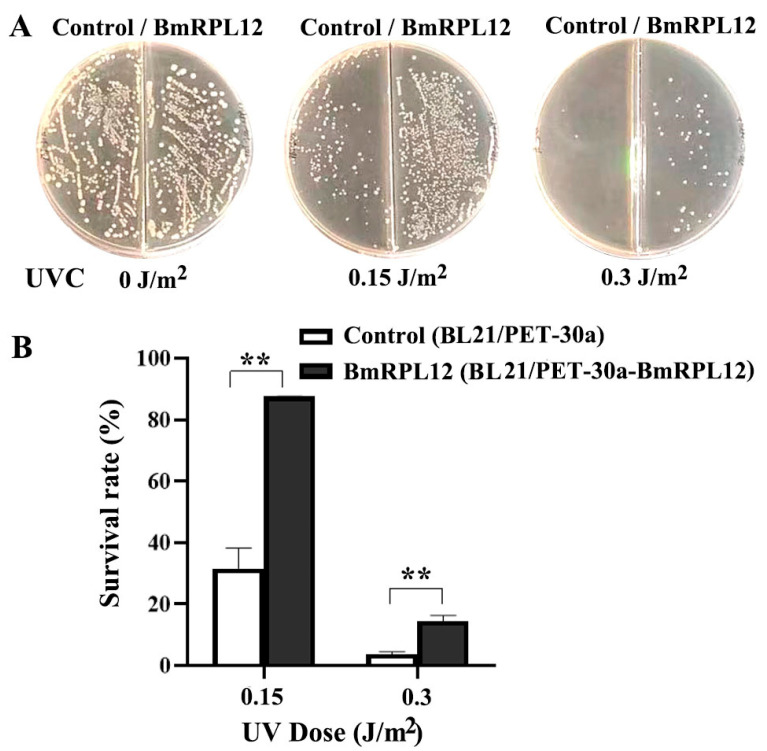
Effects of BmRPL12 on the UV sensitivity of *E. coli*. (**A**) The growth of BmRPL12-expressing BL21 cells after UV radiation. The BmRPL12-expressing cells and control cells, respectively, were spread onto LB agar plates and exposed to different doses of UVC. After overnight cultivation, the number of colonies was photographed and counted. (**B**) UV survival rate analysis of BmRPL12-expressing BL21 cells. The percentage survival was calculated as the ratio of UV-treated to untreated cells. Error bars indicated the mean ± standard deviation from three independent experiments. ** indicates a statistically significant difference (*p* < 0.01).

The UV survival rate of *E. coli* expressing BmRPL12 was analyzed to assess whether BmRPL12 was related to UV damage repair. The results showed that the UV survival rate of *E. coli* expressing BmRPL12 was significantly higher than that of the control group, suggesting that BmRPL12 contributed to the UV damage repair (Figure 2).

### 3.3. BmRPL12 Was Mainly Localized in the Cytoplasm of BmN Cells

**Figure 3:**
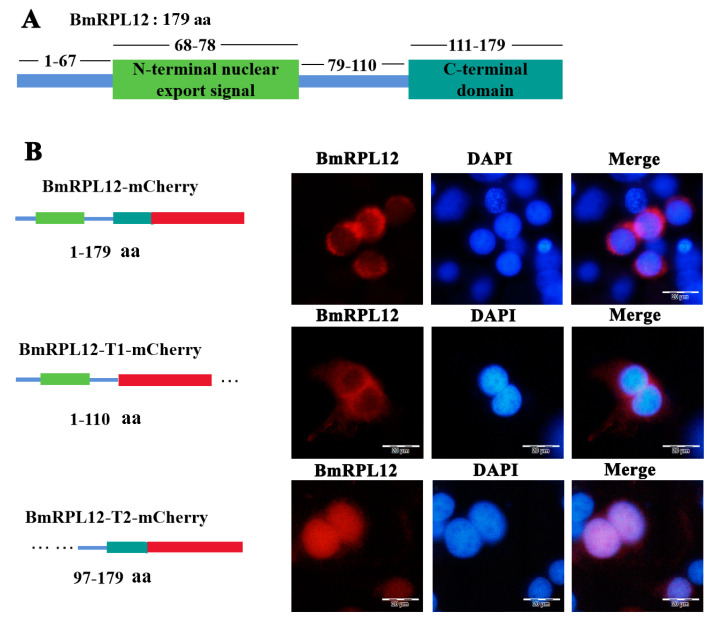
Fluorescence microscope observation of full-length BmRPL12 and truncated BmRPL12. The BmN cells were transfected with the recombinant plasmids to express mCherry-tagged BmRPL12 proteins (**A**). From top to bottom (**B**): the localization of BmRPL12-mCherry, BmRPL12-T1-mCherry, BmRPL12-T2-mCherry. Full-length BmRPL12 protein (1–179 aa), truncated BmRPL12 protein (T1, 1–110 aa, missing the conservative C-terminal sequence), and truncated BmRPL12 protein (T2, 97–179 aa, missing the N-terminal nuclear export signal). A mCherry tag (red stick) was fused to the C terminus of BmRPL12 mutants to follow BmRPL12 localization.

BmN cells were transfected with the recombinant plasmids to express mCherry-tagged BmRPL12 fusion proteins. The results showed that BmRPL12 and BmRPL12-T1 (missing the conservative C-terminal sequence) were mainly localized in the cytoplasm of BmN cells. However, BmRPL12-T2 with N-terminal deletion was mainly localized in the nuclei, suggesting that the N-terminal nuclear export signal affected the localization of BmRPL12 (Figure 3).

### 3.4. Knocking Down the Expression of BmRPL12 Slowed Down the Speed of UV-Induced DNA Damage Repair

**Figure 4:**
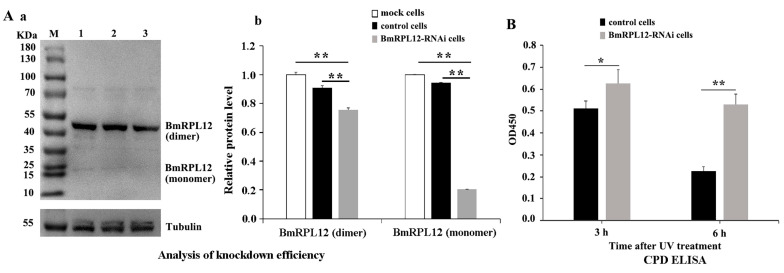
Effect of BmRPL12 on UV-induced DNA repair. (**A**) Analysis of knockdown efficiency in BmRPL12-RNAi cells. (**a**) The knockdown efficiency of BmRPL12 was determined by Western blotting. Lane 1: total proteins from mock cells; Lane 2: total proteins from negative control siRNA-transfected cells; Lane 3: total proteins from BmRPL12 siRNA-transfected cells. α-Tubulin was used as a loading control. (**b**) The western blot bands was quantified by ImageJ software to determine the knockdown efficiency of BmRPL12. (**B**) Analysis of UV-induced DNA damage in BmRPL12-RNAi cells and control cells by CPD ELISA kit. Error bars indicated the mean ± standard deviation (SD) from three independent experiments. * indicates a statistically significant difference (*p* < 0.05); ** indicates a statistically significant difference (*p* < 0.01).

To further confirm whether BmRPL12 affected UV-induced DNA damage repair, the DNA repair capability in BmRPL12-knockdown cells was analyzed. In advance, the knockdown efficiency of BmRPL12 was verified by Western blotting. ImageJ software was further used to quantify the western blot bands to determine the knockdown efficiency of BmRPL12. The results showed that the expression of BmRPL12 was inhibited in BmRPL12-RNAi cells, especially the expression level of monomer was more significantly reduced. The results also showed that BmRPL12 existed in BmN cells in both monomeric and dimeric forms, and the dimeric forms accounted for the vast majority (Figure 4A).

BmRPL12-knockdown cells and control cells (negative control siRNA-transfected cells) were exposed to 60 J/m^2^ UVC. The DNA was separately extracted at different time points to analyze UV-induced DNA damage using the CPD-ELISA kit. The results showed that the DNA damage (CPD) decreased slower in BmRPL12-knockdown cells (Figure 4B), suggesting that BmRPL12 played an important role in UV-induced DNA damage repair.

### 3.5. Reciprocal Interaction Between BmRPL12 and Bm65

**Figure 5:**
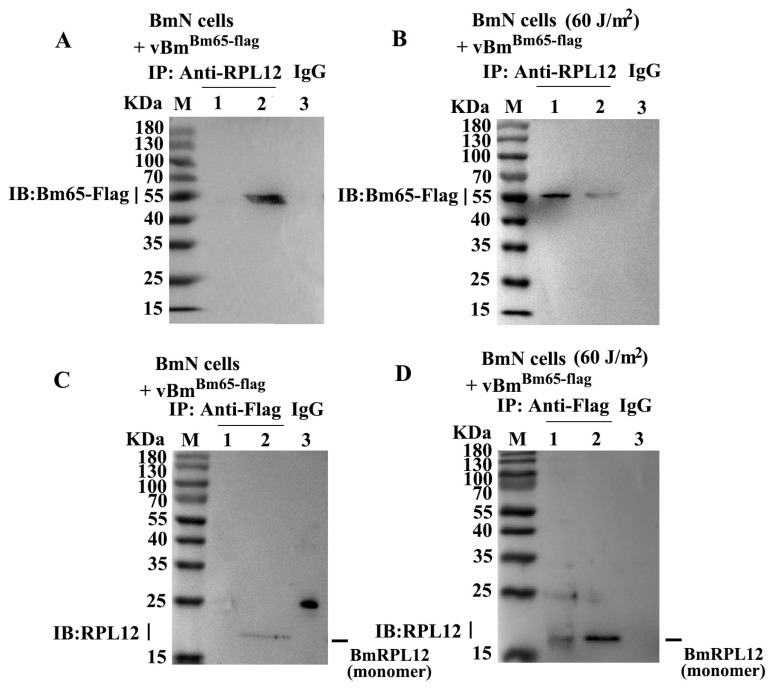
Analysis of the interaction between BmRPL12 and Bm65 by Co-IP. (**A**,**C**) Analysis of the interaction between BmRPL12 and Bm65 in vBm^Bm65-flag^-infected BmN cells (cells without UV treatment). (**B**,**D**) Analysis of the interaction between BmRPL12 and Bm65 in vBm^Bm65-flag^-infected BmN cells (cells with 60 J/m^2^ UVC treatment before infection). Lane 1: Co-IP with anti-RPL12 or anti-Flag antibodies; Lane 2: input; Lane 3: normal IgG was used as a negative IP control. The anti-Flag monoclonal antibody was used to detect Bm65-Flag, and the anti-RPL12 polyclonal antibody was used to detect BmRPL12 in Western blotting.

BmN cells were infected by vBm^Bm65-flag^ to express Bm65-Flag. The interaction between BmRPL12 and Bm65 was verified with anti-RPL12 antibodies and anti-Flag antibodies by Co-IP and Western blotting. The results showed that the binding of BmRPL12 to Bm65 was detected only in UV-treated experimental groups (the UVC-treated cells infected with vBm^Bm65-flag^) (Figure 5B,D). In the above experiment, BmRPL12 mainly existed in the form of dimers (Figure 4A). However, only monomeric BmRPL12 proteins were found to interact with Bm65 after UV radiation (Figure 5D).

### 3.6. The Colocalization of BmRPL12 and Bm65 in BmN Cells

**Figure 6:**
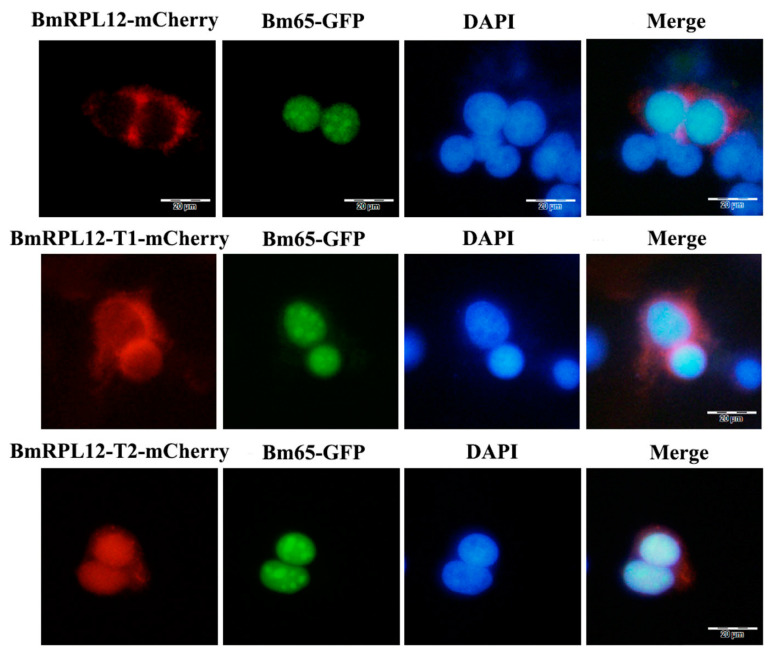
Colocalization analysis of BmRPL12 and Bm65. From top to bottom: the colocalization analysis of BmRPL12-mCherry and Bm65-GFP, the colocalization analysis of BmRPL12-T1-mCherry (1–110 aa) and Bm65-GFP, the colocalization analysis of BmRPL12-T2-mCherry (97–179 aa) and Bm65-GFP. The BmN cells were transfected with the recombinant plasmids to express mCherry-tagged BmRPL12 proteins and GFP-tagged Bm65 proteins. Truncated BmRPL12 protein (T1, 1–110 aa, missing the conservative C-terminal sequence); truncated BmRPL12 protein (T2, 9–179 aa, missing the N-terminal nuclear export signal).

The BmN cells were separately transfected with plasmids to express BmRPL12-mCherry, BmRPL12 truncated proteins, and Bm65-GFP. Fluorescence observation showed that BmRPL12, BmRPL12-T1 (missing the conservative C-terminal sequence), and BmRPL12-T2 (missing the N-terminal sequence) did not colocalized with Bm65 in the BmN cells (Figure 6). These subcellular localization results were consistent with the Co-IP results. When there was no UV radiation, BmRPL12 was unable to interact or co-located with Bm65.

### 3.7. BmRPL12 Specifically Aggregated at the UV-Induced DNA Damage Sites Only in the Presence of Bm65

**Figure 7:**
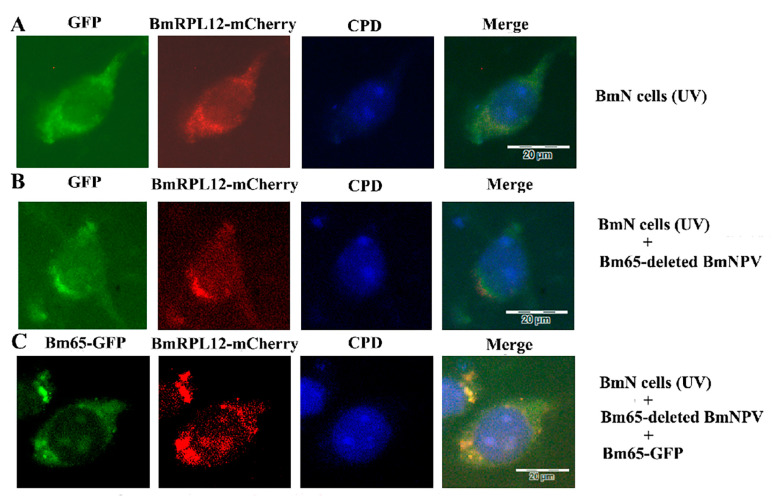
Localization analysis of BmRPL12 with UV-induced DNA damaged by immunofluorescence: (**A**) localization analysis of BmRPL12-mCherry with UV-induced DNA damage in BmN cells; (**B**) localization analysis of BmRPL12-mCherry with UV-induced DNA damage in Bm65-deleted viruses-infected cells; (**C**) localization analysis of BmRPL12-mCherry with UV-induced DNA damage in Bm65-deleted virus-infected cells (these cells were transiently transfected to express Bm65-GFP or GFP before infection). Dot-shaped blue fluorescence indicated UV-induced DNA damage (CPD). GFP was used as control.

The cells were covered with a UV-opaque polycarbonate filter membrane (5 μm pores in the membranes) and exposed to UV light to cause local DNA damage. Anti-CPD monoclonal antibodies and corresponding fluorescent secondary antibodies were used to detect UV-induced DNA damage. Fluorescence observation showed that BmRPL12 could not specifically aggregate at UV-induced DNA damage sites, suggesting that BmRPL12 was unable directly participate in UV-induced DNA damage repair by binding to DNA (Figure 7A).

When UV-damaged cells were infected with Bm65-deleted viruses (vBm^Bm65KO^), BmRPL12 still could not aggregate at the UV-induced DNA damage sites (Figure 7B). When vBm^Bm65KO^-infected cells express Bm65 proteins through transfection, BmRPL12 was able to co-localize with Bm65 at the UV-induced DNA damage sites (Figure 7C), suggesting that BmRPL12 specifically aggregated at the UV-induced DNA damage sites only through interacting with Bm65.

## 4. Discussion

Currently, an increasing number of ribosomal proteins have been discovered to be multi-functional proteins with various extra-ribosomal functions. Some ribosomal proteins have been reported to be associated with DNA damage repair. rpS3 is found to play a role in DNA damage repair by enhancing XPD helicase function [19]. In addition, rpS3 has endonuclease activity for DNA repair [20]. RPL6 is found to be recruited to regulate DNA damage response at DNA damage sites [21]. It is also reported that RPL18 is involved in the adaptation to UV-B in *Chlamydomonas reinhardtii* [22].

The expression of ribosomal proteins is often stable. Ribosomal protein genes RpS3 and RpL13A in *Tribolium castaneum* showed high stability across all UVB irradiation time points for RT-qPCR analysis and were suitable as a reference gene [23]. RPL12 is also used as a reference gene [24,25]. In this study, *E. coli* expressing BmRPL12 was proven to have stronger resistance to UV radiation. The subcellular localization results showed that BmRPL12 was mainly located in the cytoplasm, and the N-terminal nuclear export signal was crucial for the protein localization. BmRPL12 was unable to specifically locate at the UV-induced DNA damage sites. UV-induced DNA damage repair was further found to slow down in BmRPL12-knockdown cells. The above results indicated that BmRPL12 proteins was unable to directly participate in UV-induced DNA damage repair at the DNA damage sites but indirectly affected the repair efficiency. Furthermore, we found that BmRPL12 proteins mainly existed in dimeric forms. Dimeric proteins are the most abundant form of protein assembly and play a critical role in many biological processes. Protein dimerization can regulate a series of biological processes, such as enzyme activation, signal transduction, and pathogenic pathways [26]. Therefore, we speculate that BmRPL12 proteins mainly play roles in the form of dimers.

Viruses and hosts influence and compete with each other over a long period of evolution. During viral infection, often there is an interplay between host and viral proteins and usually viral proteins usurp host proteins to aid the viral life cycle. Viral infection also causes changes in the subcellular localization of some host proteins [27]. Our previous research found that Bm65 was an important UV endonuclease and beneficial for DNA damage repair [13,14,28]. In addition, other genes in BmNPV are also found to be important for DNA damage repair or nucleotide formation, such as vp39 and other viral proteins that play crucial roles in late phenotypes [29]. In this study, we further confirmed that BmRPL12 only interacted with Bm65 in monomeric form after UV radiation, and that BmRPL12 aggregated at the UV-induced DNA damage sites only in the presence of Bm65. We will continue to investigate whether BmRPL12 regulates the UV endonuclease activity of Bm65 through interaction with Bm65 in the future.

**Figure 8:**
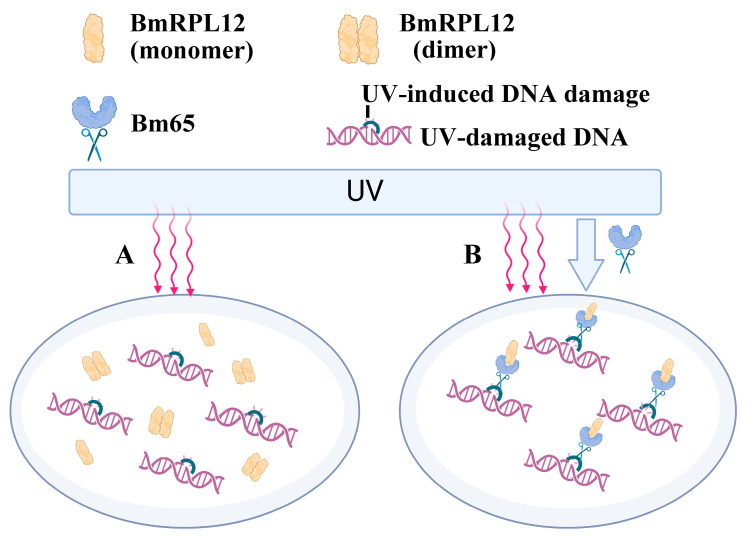
Schematic diagram showing the possible mechanisms of BmRPL12 participating in UV-induced DNA damage repair: (**A**) BmRPL12 indirectly participated in UV-induced DNA damage repair; (**B**) BmRPL12 directly participated in DNA damage repair at the UV-induced DNA damage sites through interactions with Bm65 in the form of monomers after viral infection.

In summary, after UV radiation, virus protein Bm65 selectively interacted with monomeric form of BmRPL12 protein and altered its localization. It was speculated that BmRPL12 indirectly participated in UV-induced DNA damage repair in the form of dimers but also directly participated in DNA damage repair through interactions with Bm65 in the form of monomers (Figure 8). Further experiments are needed to elucidate the specific molecular mechanism of BmRPL12-Bm65 regulation of UV-induced DNA damage repair and also investigate the relationship between BmRPL12 function and its dimerization.

## Figures and Tables

**Table 1:** All primers used in the study.

Primer Name	Primer Sequence (5′→3′)	Enzyme Digestion Sites
BmRPL12-F	CGGAATTCATGAACGGAATCAGAATAATTAAAAAT	*EcoR* I
BmRPL12-R	CGCTCGAGTTCAATTTCTATAATGGCTCCAACTTT	*Xho* I
BmRPL12-gfp-F	CGGAATTCATGAACGGAATCAGAATAATTAAAAAT	*EcoR* I
BmRPL12-gfp-R	CGCTCGAGTTCAATTTCTATAATGGCTCCAACTTT	*Xho* I
BmRPL12-mCherry-F	CGGAATTCATGAACGGAATCAGAATAATTAAAAAT	*Pst* I
BmRPL12-mCherry-R	CGCTCGAGTTCAATTTCTATAATGGCTCCAACTTT	*Xho* I
BmRPL12-T2-mCherry-F	ATGAATTCATGGTTGATGAAGAAGAGGCAGCCCCGAAA	*EcoR* I
BmRPL12-T1-mCherry-R	ATCTCGAGACTGGTCTTCACAGCTTTCGGGGCTGC	*Xho* I
BmRPL12 siRNA	GUGGCGAUCUUUAUCAAGATT UCUUGAUAAAGAUCGCCACTT	
siRNA (negative control)	UUCUCCGAACGUGUCACGUTT ACGUGACACGUUCGGAGAATT	

Note: Underlined letters indicate restriction enzyme digestion sites.

## Data Availability

The original contributions presented in the study are included in the article. Requests for reagents developed in this work such as cell lines and plasmids as well as raw images can be directed to the corresponding author.
